# Perineural resiniferatoxin selectively inhibits inflammatory hyperalgesia

**DOI:** 10.1186/1744-8069-4-3

**Published:** 2008-01-16

**Authors:** John K Neubert, Andrew J Mannes, Laszlo J Karai, Alan C Jenkins, Lanel Zawatski, Mones Abu-Asab, Michael J Iadarola

**Affiliations:** 1 College of Dentistry Department of Orthodontics, University of Florida, Gainesville, FL, USA; 2 College of Medicine Department of Neuroscience, University of Florida, Gainesville, FL, USA; 3 Evelyn F. and William L. McKnight Brain Institute, University of Florida, Gainesville, FL, USA; 4 Neurobiology and Pain Therapeutics Section, Laboratory of Sensory Biology, National Institute of Dental and Craniofacial Research, National Institutes of Health, Bethesda, MD, USA; 5 Section of Ultrastructural Pathology, Laboratory of Pathology, National Cancer Institute, National Institutes of Health, Bethesda, MD, USA

## Abstract

Resiniferatoxin (RTX) is an ultrapotent capsaicin analog that binds to the transient receptor potential channel, vanilloid subfamily member 1 (TRPV1). There is a large body of evidence supporting a role for TRPV1 in noxious-mediated and inflammatory hyperalgesic responses. In this study, we evaluated low, graded, doses of perineural RTX as a method for regional pain control. We hypothesized that this approach can provide long-term, but reversible, blockade of a portion of nociceptive afferent fibers within peripheral nerves when given at a site remote from the neuronal perikarya in the dorsal root ganglia. Following perineural RTX application to the sciatic nerve, we demonstrated a significant inhibition of inflammatory nociception that was dose- and time-dependent. At the same time, treated animals maintained normal proprioceptive sensations and motor control, and other nociceptive responses were largely unaffected. Using a range of mechanical and thermal algesic tests, we found that the most sensitive measure following perineural RTX administration was inhibition of inflammatory hyperalgesia. Recovery studies showed that physiologic sensory function could return as early as two weeks post-RTX treatment, however, immunohistochemical examination of the DRG revealed a partial, but significant reduction in the number of the TRPV1-positive neurons. We propose that this method could represent a beneficial treatment for a range of chronic pain problems, including neuropathic and inflammatory pain not responding to other therapies.

## Introduction

Resiniferatoxin (RTX), an ultrapotent capsaicin analog, is a vanilloid agonist that binds to the transient receptor potential channel, vanilloid subfamily member 1 (TRPV1), a non-selective cation ion channel expressed in small- and medium-sized neurons in sensory ganglia [1-3]. Evidence from TRPV1 gene deletion studies supports a role for TRPV1 in noxious thermal, chemical, and inflammatory hyperalgesic responses [4,5]. Behaviorally, long duration noxious heat analgesia can be induced by subcutaneous RTX administration [6-9]. These data reinforce the long-recognized idea that TRPV1 has an important role in inflammation and pain and thus TRPV1 has become a target for analgesic drug development [10-16].

Vanilloids can trigger a Na+/Ca^2+ ^flux in axons of primary cultured dorsal root ganglia (DRG) neurons [17]. We have observed that when RTX is administered in close proximity to the cell body of TRPV1-positive neurons (*i.e*., intra-ganglionic), it induces a Ca^2+^-excitotoxicity and subsequent permanent neuronal cell deletion [18,19]. This route of application has been proposed as a treatment for pain in advanced metastatic disease [9,20,21]; however, a reversible approach for achieving analgesia represents an alternative route with certain advantages for pain control in non-malignant situations. Based on prior studies using peripheral application of vanilloid agonists such as capsaicin and RTX [8,22-25], we hypothesized that perineural application of RTX may temporarily block transmission in TRPV1-containing axons. The percutaneous perineural approach is proposed to broadly target a specific nerve and its' peripheral receptive fields to produce a selective antinociceptive action while maintaining other somatic and proprioceptive sensations conveyed by large diameter TRPV1-negative axons. By modifying transmission through the peripheral nerve, this method can provide analgesia in a wide range of chronic pain problems. Perineural application has recently been explored using high doses of RTX (1500 ng) to eliminate thermal and inflammatory heat sensitivity and reduce motor reaction threshold responses to pressure [24]. However, in the clinical setting, exploration of the lower range of doses needs to be considered in order to determine the minimum effective dose for analgesia that reduces or eliminates potential side effects. In the present study, we investigate the efficacy of lower doses of RTX and evaluate the temporal recovery of function by using perineural RTX against acute pain challenges and evaluate its effectiveness in preemptively blocking inflammation-induced hyperalgesia.

## Methods

All procedures complied with the guidelines of the Committee for Research and Ethical Issues of IASP and the Institutional Animal Care and Use Committee of the National Institute of Dental and Craniofacial Research, National Institutes of Health.

### RTX or vehicle application

**Figure 1:**
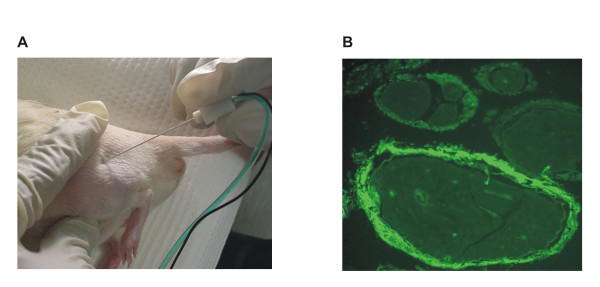
**Locating the sciatic nerve for electrically-stimulated percutaneous, perineural RTX injection**. Note finger palpation locations and direction of the needle (*see Methods*). Fluorescein dye injection (50 μl, 1 μg/μl) using this technique demonstrates the distribution of dye-containing fluid within the perineurium of the nerve (**B**).

Adult male Sprague Dawley rats (200–300 gm) were anesthetized (2.5% isoflurane, USP, inhalation), the outer portion of rear left thigh shaved, and the area sequentially disinfected with Betadine (10% povidone-iodine, Purdue Frederick Co, Stamford, CT) and alcohol (70% isopropanol). The femur was palpated at the superior (greater trochanter) and inferior aspects (condyle) of the bone and a sterile Stimuplex insulated needle (Stim-A25, 24 gauge × 1 inch, B. Braun Medical Inc, Bethlehem, PA) was inserted approximately 5 mm through the skin, 1 cm ventral and 1 cm rostral to the greater trochanter (Fig. 1A). The tip of the needle, with active electrical stimulation, was advanced toward the left sciatic nerve until a distal dorsal flexion of the hindpaw was elicited at 0.1 mA (1 Hz pulses). RTX (N = 46, 25 – 250 ng, LC Laboratories, Woburn, MA), or vehicle (N = 16, 0.25% Tween 80 in phosphate buffered saline (PBS), 0.05% ascorbic acid) was injected in a volume of 50 μl. Note that the vehicle used was the same as the highest dose of RTX (250 ng). Immediate loss of paw flexion was noted following the injection, even with continued stimulation, as the injection fluid shunted the stimulus to the nerve, thereby confirming a perineural distribution of the fluid. In preliminary studies using PBS, larger currents (0.2 – 0.4 mA) could produce dorsal flexion from at areas distant from the sciatic nerve, however, the flexion response was not lost when fluid was injected at this distant site.

While the sciatic nerve innervates the majority of the hindpaw, a small part of the medial plantar surface of the hindpaw is also innervated by the saphenous nerve and the stimulus can overlap to this receptive field. Therefore the saphenous nerve was additionally injected in a subset of animals (N = 15) to evaluate the effects of RTX when applied to both the sciatic and saphenous nerves. Following percutaneous sciatic nerve injection (see above), a 0.5 cm incision was made into the medial aspect of the leg at the mid-thigh level and RTX (250 ng, 50 μl) was injected directly onto the saphenous nerve. A single Vicryl resorbable (4-0, Ethicon) suture was used to close the wound and topical triple antibiotic cream (Neomycin, Polymyxin B, and Bacitracin ointment, USP) was applied.

To further assess the efficacy of the percutaneous approach, we examined a group of animals (N = 10) that received RTX via a direct, open surgical procedure. Animals were anesthetized, surgically prepared as described above, and a 1 cm incision was made through the skin at the mid-thigh portion of the hind limb. The underlying gluteus superficialis muscles were bluntly dissected and the sciatic nerve exposed. The overlying fascia was carefully removed and RTX (250 ng, 50 μl) was bathed over 1 cm of the nerve for 5 min, after which the wound was closed with Vicryl resorbable (4-0, Ethicon) sutures and the animals were allowed to recover. Note that the RTX solution was not washed off prior to suturing the area to better simulate the exposure produced via the percutaneous injection method.

### Fluorescein imaging

To determine the flow and uptake of fluid to and around the sciatic nerve, Fluorescein-5-isothiocyanate (FITC, 'Isomer I', 0.05 mg in 50 μl PBS, Molecular Probes, Eugene, OR) was injected into a subset of animals (N = 2) to verify targeting of the sciatic nerve (Fig. 1B). Animals were sacrificed (CO_2 _inhalation) and the injected sciatic nerve was dissected free, cut in cross-section (5 μm), formalin fixed and paraffin embedded. Sections were visualized under an Olympus BX-60 microscope equipped with a RT-Slider CCD camera (Diagnostic Instruments Inc. Burroughs, MI), using the appropriate filters and the Spot Advance software.

### Behavioral Assessments

Development of heat hyperalgesia and analgesia to radiant thermal stimulation was determined by placing unrestrained animals under a small plastic box (9 × 6 × 6 in) on an elevated clear glass platform, as described previously [26,27]. Animals were allowed to habituate for at least 5 min prior to testing. Testing was performed prior to injection, and at various time points (2.5 hrs, 1 day, 1 week, 2 weeks, 1 month, 3 months, 6 months) following the nerve injection and 2.5 hrs after hindpaw carrageenan injection. Statistical analyses (one-way and two-way repeated measures ANOVA with Scheffe post-hoc analysis) were used to compare baseline withdrawal latency times to post-treatment times and treated versus untreated values.

Sensitivity to mechanical stimuli was evaluated using an electronic Von Frey anesthesiometer (Model 1601, IITC Inc, Woodland Hills, CA). Following placement of animals under the plastic box on an elevated open-grated surface and habituation (5 min), the probe tip was applied perpendicularly to the ventral paw surface and pressure was applied until the animal withdrew its paw. The average force (gm) to elicit paw withdrawal was calculated from three iterations (5 min between each trial). Raw threshold values were normalized to the initial baseline values (base) and time and treatment effects were analyzed using a Mann-Whitney rank sum test or Kruskal-Wallis ANOVA (P < 0.05 significance). Effects on gross behaviors (grooming, limb guarding, and licking) following RTX injection were also assessed.

Gross motor impairment was evaluated using a rotarod apparatus (Model 7650, Ugo Basile, Comerio VA, Italy), as described previously [8]. The time (sec) required for the animal to fall off an accelerating rotating wheel drum (4–40 r.p.m. in 5 min) was recorded and time and treatment (RTX or vehicle) effects were analyzed using one-way ANOVA and two-way repeated measures ANOVA (p < 0.05).

### Inflammation and paw thickness measurements

Carrageenan (6 mg, Sigma, St Louis, MO) was injected (150 μl, i.pl.) into the mid-plantar surface of the hindpaw of unanesthetized rats as described previously [26]. Inflammation was initiated at the following post-injection (RTX or vehicle) time points: 1 day; 1 week; 2 weeks; 1 month; 3 months; and 6 months. Paw thickness was also measured at the mid-hindpaw level before and after carrageenan inflammation for a subset of animals pretreated with either RTX (N = 5) or vehicle (N = 5); the difference (mm) from pre- to post-inflammation was calculated.

### Neurogenic inflammation

Animals were assessed 1 week after perineural RTX or vehicle injection. Following anesthesia (pentobarbital, 50 mg/kg, i.p.), the hair from the right and left rear legs was completely removed using a depilatory cream (Nair, Carter-Wallace, Inc, Cranbury, NJ). A PE10 catheter was placed in the jugular vein and Evans Blue (EB, Sigma, St. Louis, MO) was infused over 10 min (30 mg/kg; 2% solution, i.v). Capsaicin cream (1%) was liberally and evenly applied to the legs and to the dorsal and plantar surfaces of both hindpaws and digital pictures were captured (Sony MVC-FD100 FD Mavica digital still camera).

### Immunohistochemistry

Animals were deeply anesthetized (pentobarbital, 100 mg/kg, i.p.) 31 days post-RTX injection and fixed by transcardial perfusion with cold phosphate buffered saline (PBS) followed by cold Streck's Tissue Fixative (STF). Right and left lumbar 4–5 (L4-L5) dorsal root ganglia (DRG) were dissected and placed in STF for post-fixing. Samples were paraffin processed and sections were cut (7 μm) and mounted on slides. Following deparaffinization and epitope unmasking with Target Retrieval Solution (S1700, Dako, Carpinteria, CA) at 95°C for 20 min, sections were blocked with 10% normal goat serum (S-1000, Vector Laboratories, Inc., Burlingame, CA) and incubated overnight at 4°C with the TRPV1 primary antibody (1:10,000, Oncogene, #PC420). Antibody detection was performed using the Vectastain Elite Rabbit IgG and Peroxidase Substrate Kits (SK-4700 and SK-4100, respectively, Vector Laboratories, Inc., Burlingame, CA). Sites of peroxidase activity were visualized using 3,3'-diaminobenzidene tetrahydrochloride (DAB). Control specimens for assessment of non-specific binding were processed in an identical way except for omission of the primary antibody. Histological sections were visualized with an Olympus BX 60 microscope, equipped with a RT Slider CCD camera and processed with the Spot Advanced software.

A total of N = 7 animals were used for the histological analysis: N = 5 animals had RTX (250 ng, 50 μl) applied to the left sciatic nerve and vehicle (0.25% Tween 80 in PBS, 0.05% ascorbic acid, 50 μl) applied to the right sciatic nerve. A total of N = 10 DRG were examined for each treatment from these 5 animals and a blinded observer chose non-adjacent sections (separated by > 100 μm) from different levels of the DRG for each treatment group to give a final N = 15 sections/group. The remaining N = 2 untreated naïve animals had 2 DRGs harvested from each, with a total of N = 6 sections counted. The number of TRPV1 immunoreactive and non-immunoreactive cell bodies within a rectangular reticule were counted by visual inspection (10× magnification); the ratio of TRPV1 to the total number of cells was calculated and comparisons between treatment groups (RTX, vehicle, no treatment) were performed using a one-way ANOVA and Scheffe post-hoc test, with significance set at a level of P < 0.05.

## Results

### Behavioral observations and effects on inflammatory edema

We were interested in assessing general behavioral and anti-inflammatory effects of perineural RTX. Following injection (RTX or vehicle) and recovery from anesthesia (< 10 min), animals did not display nociceptive behaviors, such as licking, guarding, which we had observed in previous studies upon injection into the hindpaw (Neubert 2003). We also never observed abnormal behaviors such as autotomy in any of the animals, such as seen with complete sciatic denervation [28]. Perineural RTX did not affect paw edema following carrageenan inflammation (change in hindpaw size was 6.2 ± 0.3 mm for RTX, 6.0 ± 0.2 mm for vehicle). These data indicate that the doses of RTX used for analgesia did not inhibit the inflammatory stimulus provided by the carrageenan injection.

### RTX blocks inflammatory heat hyperalgesia when applied directly to the sciatic nerve in a time and dose dependent fashion

**Figure 2:**
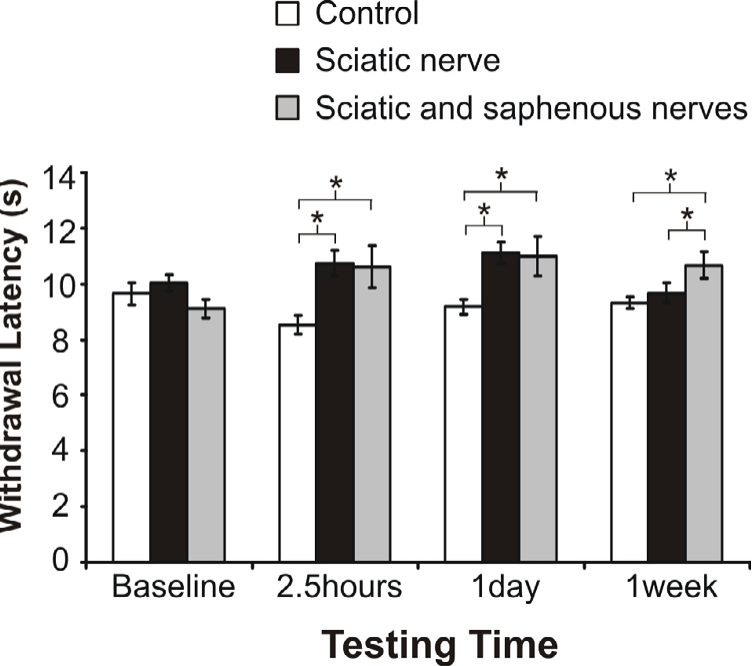
**Percutaneous application of RTX to the sciatic nerve and sciatic plus saphenous nerves minimally effects response to heat stimuli**. RTX (250 ng, 50 μl) application to only the sciatic nerve produced a transient change in heat withdrawal latency with respect to testing time (*P < 0.05, 1 way ANOVA, Scheffe post-hoc analysis), as compared to the baseline. Additional application of RTX to the saphenous nerve produced a modest increase in heat latency, but this effect was not significantly different, as compared to the sciatic group. The "sciatic nerve" group refers to animals treated only with percutaneous RTX (N = 24) and the "sciatic and saphenous nerves" group refers to animals that were injected percutaneously near the sciatic nerve and had an open field injection around the saphenous nerve (N = 15). The "control group" represents the uninjected right hindpaw. There was no difference between contralateral controls for the sciatic nerve group (N = 24) and the sciatic/saphenous group (N = 15), therefore these groups were combined (N = 39) for subsequent comparisons.

**Figure 3:**
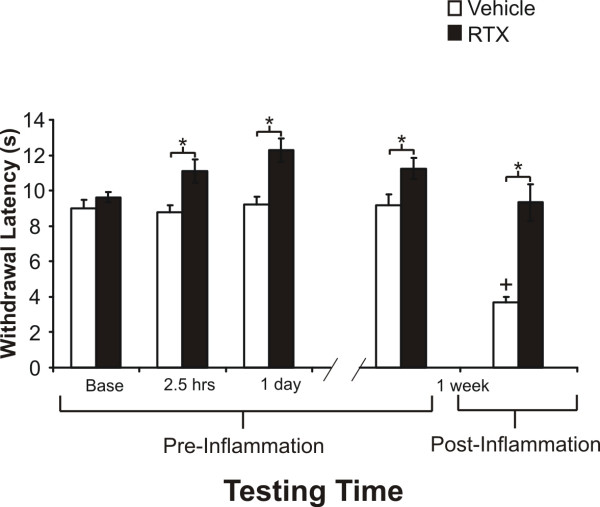
**RTX blocks inflammatory heat hyperalgesia when applied directly to the sciatic nerve**. Both percutaneous and open field injection of RTX (250 ng, 50 μl) inhibited inflammatory heat hyperalgesia following carrageenan injection (6 mg, 150 μl). There was no significant difference in latency time regarding RTX application method (*i.e*. percutaneously vs. direct open field application); therefore data were pooled (N = 15). However, for this set of animals, there was a significant increase in normal heat latency as compared to vehicle treated animals (*P < 0.05, 1 way ANOVA, Scheffe post-hoc analysis). Animals pretreated with vehicle (0.25% Tween-80, PBS 50 μl, N = 10) had a significant decrease in their heat withdrawal latency (^+^P < 0.05, 1 way RM-ANOVA) following induction of inflammation.

RTX produced a small but significant increase (range = 1–3 sec net change) in hind paw heat withdrawal latency ipsilaterally when injected perineurally around the sciatic nerve, as compared to contralateral hindpaw, which we used as the control, or vehicle treated animals (Figs. 2 and 3). This increase was also observed when animals were injected at both the saphenous and sciatic nerve sites, with a significant increase noted at 1 week post-injection as compared to the single sciatic nerve injection (Fig. 2). In contrast to the effect on acute heat sensation, there was a more substantial effect for perineural (percutaneous or open surgical) RTX application on inhibiting heat hyperalgesia following inflammation, as compared to animals treated with vehicle and to pre-inflammation testing times (Fig. 3). Animals treated with vehicle had a statistically significant decrease in latency following inflammation (P < 0.01) compared to the RTX treated group (Fig. 3). There were no significant differences in latency times regarding method of RTX application to the nerve, *i.e*. percutaneous injection versus the open surgical application (8.4 ± 2.1 sec versus 10.0 ± 1.0 sec, respectively). Therefore, the double site injection was not pursued further. These data indicate that even animals that display only a modest increase in thermal latency under non-inflammatory conditions, exhibit a strong anti-hyperalgesic effect during carrageenan inflammation. Since this endpoint was the most sensitive parameter, we used it to follow the time course of recovery from the RTX analgesic effect.

**Figure 4:**
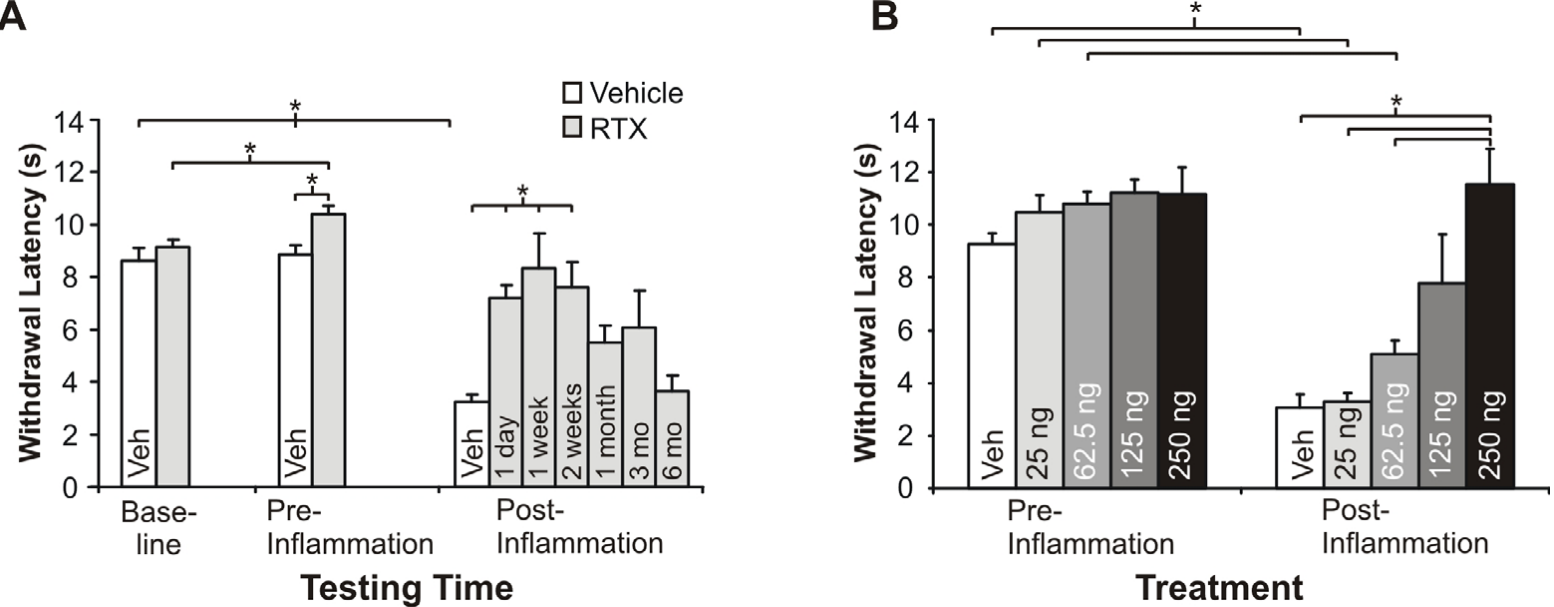
**Percutaneous RTX produces long-lasting, reversible inflammatory heat hyperalgesic inhibition (A) in a dose-dependent fashion (B)**. Inflammatory heat hyperalgesia is significantly inhibited 1 day (N = 8), 1 week (N = 6), and 2 weeks (N = 5) following application of RTX (250 ng, 50 μl) to the sciatic nerve (*P < 0.05, 1 way ANOVA). An intermediate response was seen at 1 month (N = 8), and at 3 months (N = 8). Complete recovery with a normal heat withdrawal response following inflammation was observed 6 months (N = 11) following treatment, indicating that the effect of RTX had reversed. The dose-response results (B) indicate that ≥ 125 ng is necessary for a significant anti-inflammatory effect, as compared to Pre-Inflammation values and to vehicle (P < 0.05). Note that the Pre-inflammation testing point represents the same testing day that the animal was to be inflamed. There was no difference between groups treated with RTX (N = 46); therefore data were pooled for the baseline (Baseline) and Pre-inflammation testing sessions. Animals treated with vehicle (N = 16) were also pooled in the Baseline, Pre-inflammation, and Post-inflammation groups for comparison.

Perineural application of RTX (250 ng, 50 μl) to the sciatic nerve produced a significant inhibition of heat hyperalgesia following carrageenan-induced inflammation as soon as 1 day post-injection and lasting for 2 weeks (Fig. 4, P < 0.05, 1 way RM ANOVA). At later times, using a repeated measure ANOVA, no statistically significant block of hyperalgesia was seen at 1, 3, and 6 months post-injection. These data show that recovery of inflammatory heat hyperalgesia occurs between 2 weeks and 6 months. This is consistent with the idea that the behavioral analgesic effects of perineural RTX are reversible (Fig. 4A). In vehicle treated rats there was a significant reduction in the heat withdrawal latency compared to pre-inflammation responses and to the 1 day, 1 week, and 2 weeks RTX treated groups. We tested for an effect of vehicle as well at 48 hrs and 1 week following perineural vehicle injection, but prior to carrageenan inflammation, and there was no change in comparison to the contralateral side, therefore the groups (N = 16) are combined in Fig. 4A.

RTX inhibited inflammatory heat hyperalgesia in a dose-dependent fashion, with the effective dose being ≥ 125 ng (Fig. 4B). The lower doses did not significantly block inflammatory hyperalgesia, as compared to vehicle. For the 250 ng group following inflammation, there was no significant difference compared to pre-inflammation values, and these groups had significantly higher values as compared to doses of 62.5 ng and lower. We chose to use the 250 ng dose throughout the rest of the study to minimize variability in responses at the lower effective dose (125 ng). There were no significant differences between any of the doses and vehicle on pre-inflammatory heat withdrawal latency for these sets of animals (Fig. 4B).

### Mechanical sensitivity and rotarod responsiveness

**Figure 5:**
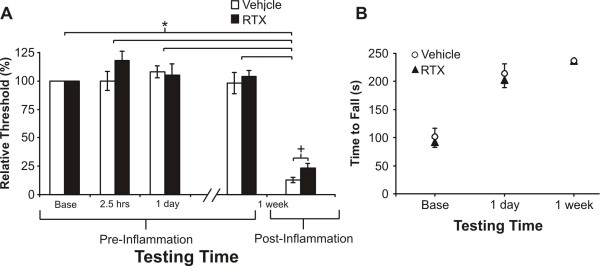
**RTX does not affect normal mechanical sensitivity or rotarod performance**. Withdrawal force latencies were similar for vehicle and RTX treated animals except after inflammation, where there was a significant difference (*P < 0.05, Mann-Whitney rank sum test) between the two groups (**A**). Data presented as a median normalized threshold (bars: interquartile range). Pre-inflammation values for both vehicle and RTX-treated groups were all significant versus the post-inflammation value (Krukal-Wallis ANOVA, P < 0.05). Values are represented by a median normalized threshold and the interquartile range is denoted by the bars. Rotarod performance was identical for both groups (**B**).

There were no differences in response to von Frey mechanical stimuli following RTX or vehicle injection (Fig. 5A). At the 1-week time point, carrageenan was given and testing was performed 2.5 h later (when the inflammation was well developed). In both vehicle and RTX treated groups, we observed a pronounced mechanical hyperalgesia, in comparison to baseline or the other testing sessions. The route of RTX administration (percutaneous or open surgical procedure) made no difference in the results, therefore these groups were pooled. After inflammation, there was a small but significant analgesic effect of RTX as compared to vehicle, but this did not result in a substantial reversal of inflammatory mechanical hyperalgesia as both the vehicle and RTX treated groups had significantly (P < 0.05) lower thresholds for withdrawal following inflammation compared to the prior, pre-inflammation testing sessions.

Rotarod results demonstrate that there were no significant differences between animals that received vehicle or RTX perineurally next to the sciatic nerve when comparing the duration (sec) the animals remained on the accelerating rotating rod (Fig. 5B). Moreover, the increase in rotarod performance over the testing times was similar for both groups, suggesting that comprehensive or delayed motor problems did not develop. Note that data derived from the accelerating rod test provides a sensitive measure for minor coordination problems since it forces both control and treated rats to perform to the point of failure. Deficits that the rat can compensate for in a slowly moving rod are revealed using the accelerating paradigm.

### Perineural RTX partially depletes TRPV1- immunopositive cells in sensory ganglia

**Figure 6:**
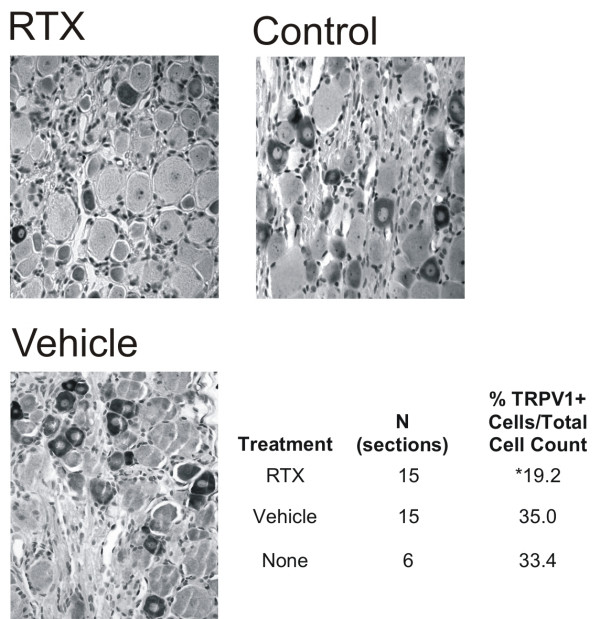
**Dorsal root gangliaTRPV1 immunopositive cells are eliminated following perineural RTX treatment**. Lumbar dorsal root ganglia sections (7 μm, paraffin-embedded) were stained for TRPV1 following RTX, vehicle or no treatment of the sciatic nerve. There was a significant difference in the number of TRPV1 positively stained cells in the RTX-treated animals, as compared to vehicle and untreated animals (**Table inset**).

RTX is known to kill cell bodies of TRPV1-positive neurons when applied a directly into sensory ganglia, producing a permanent analgesic state [9,20]. Therefore we explored whether the axonally directed, spatially remote application of RTX used here would preserve the integrity of the neuronal cell bodies in the DRG. To test this, TRPV1-positive immunoreactive (TRPV1-IR) cells were counted in the dorsal root ganglia corresponding to the sciatic nerve innervation from the RTX-treated side, vehicle-treated side, and non-treated control rats. There was a significant decrease (F_(2,35) _= 17.95, P < 0.05) in the lumbar (L4-5) DRG for the TRPV1-IR/total cells ratio 1 month after perineural RTX application (250 ng), as compared to perineural vehicle application or naïve animals (Fig. 6).

### RTX blocks neurogenic inflammation (Fig. 7)

**Figure 7:**
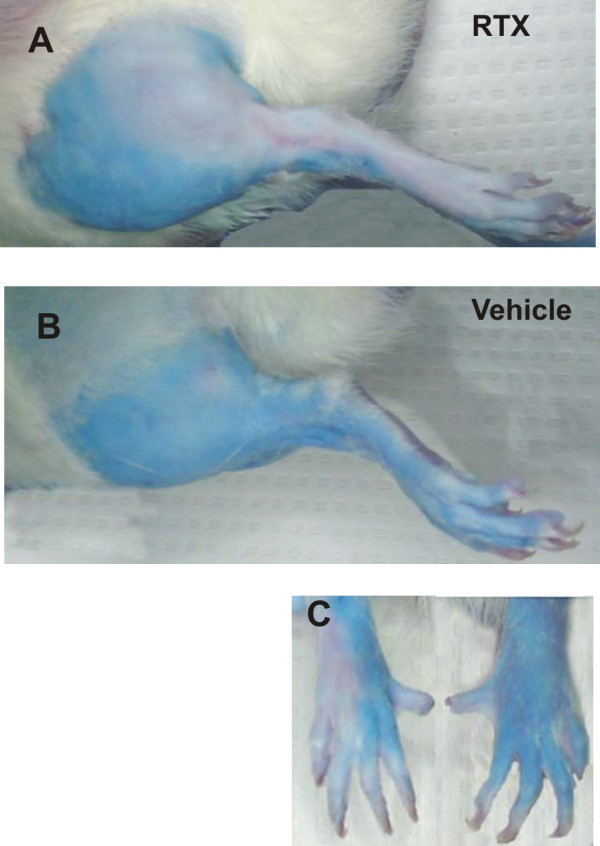
**RTX blocks capsaicin-induced efferent neurogenic inflammation**. Following Evans blue injection (i.v., 30 mg/kg, 2% solution), a 1% capsaicin cream was liberally applied to the shaved hind legs and paws. Areas of neurogenic-induced plasma extravasation are seen as blue. Note the delineation between the blue area (positive for plasma extravasation) versus the white skin (negative for plasma extravasation) for the RTX treated side (**A, C**) and extravasation on only the three medial toes for the RTX-treated side. Perineural vehicle application did not effect plasma extravasation (**B**). These data indicate selective targeting of the sciatic nerve while preserving the saphenous division via this percutaneous injection.

RTX eliminated the plasma extravasation following neurogenic inflammation induced by topical capsaicin cream (Fig. 7). In all animals, a clear demarcation demonstrating an effect on the peroneal division of the sciatic nerve is noted on the lateral aspect of the hind leg (**A**) and on the dorsum of the lateral two toes of the hindpaw in animals treated with RTX (**C**). The medial blue extravasation indicates that the saphenous nerve was spared during the RTX treatment, therefore this region was not protected from the capsaicin-induced neurogenic inflammation. The animal illustrated is typical of seven rats tested for plasma extravasation in which only the sciatic nerve was treated.

## Discussion

The selective analgesic approach to pain control without the loss of other functions has been the focus of many laboratories. For example, targeting of sodium channels such as NaV 1.8 has led to development of antagonists in the quest for novel analgesics (Veneroni et al, Pain 2003). TRPV1 antagonists have been shown to be effective for reducing chemical, thermal, and inflammatory pain without significant motor effects (Garcia-Martiez et al PNAS 2002, Gavva et al, JPET 2005). In this study, we demonstrated that perineural application of RTX produces a dose- and time-dependent inhibition of inflammatory nociceptive processes, while maintaining normal proprioceptive sensations and motor control. Remarkably, most other pain sensations were preserved except for the change in inflammatory heat hyperalgesia. There was a small but statistically significant effect on the response to mechanical stimuli when comparing RTX-treated animals with vehicle-treated animals following inflammation (Fig. 5). However, in reference to pre-inflammatory testing sessions, the difference between RTX- and vehicle-treated animals does not appear to be substantial (Fig. 5). Additional application of RTX to the saphenous nerve did not significantly affect the anti-inflammatory hyperalgesia response as compared to application to the sciatic nerve alone, although application to both the saphenous and sciatic nerves did increase the normal thermal latency (Fig. 2). However, sciatic perineural application may be sufficient for producing regional effects while maintaining normal thermal, mechanical, and proprioceptive sensations. We demonstrate a blockade of neurogenic inflammation mediated by the sciatic nerve with this percutaneous approach (Fig. 7) based on plasma extravasation in peripheral receptive fields. We observed a significant reduction of inflammatory hyperalgesia which was the most sensitive nociceptive endpoint.

Jancso and Lawson demonstrated that capsaicin applied to the saphenous nerve produced a loss of approximately a third of the unmyelinated fibers compared to control nerves, while the proportion of myelinated fibers remained unchanged [29]. Additionally, this group found that perineural capsaicin application produced a reduction in the proportion of small-sized neurons in the ipsilateral DRG. Similarly, in this study, we found a significant loss of TRPV1-positive cells following perineural RTX application in the corresponding DRG one month following treatment, but based on the behavioral outcomes, larger myelinated nerve fibers appeared to be unaffected by RTX. Furthermore, even with this permanent cell loss we observed a recovery of inflammatory hyperalgesia beginning approximately 2 weeks after injection. This is consistent with several possibilities: (a) functional repair of the RTX-affected axons occurs after that time, (b) there could be a return of function due to collateral sprouting of un-injured axons into the denervated paw, (3) sensitivity of the existing axons could be enhanced. The cell loss may impact other general functions but we did track a set of animals for 6 months and these animals displayed normal behaviors throughout. This is consistent with the Karai *et al *study that demonstrated no adverse events for up to a year following intra-trigeminal ganglia injection [9]. The mechanism for functional recovery and long-term effects will be the subject of a follow-up study.

Targeting of primary afferent nociceptive transmission at the peripheral axons provides an approach for producing regionally specific therapeutic effects [9,24,25,30]. Earlier studies using capsaicin demonstrated that direct application to peripheral nerves produced transient nociceptive activity, followed by a prolonged inhibition of responses to noxious stimuli, especially heat and inhibition of neurogenic inflammation [23,31,32]. However in this context, capsaicin produced permanent impairment of a proportion of C-fibers in sensory nerves as these capsaicin treated rats continued to exhibit a deficit when tested between 3–12 months [23,33]. In contrast, we demonstrate recovery of sensitivity to inflammatory hyperalgesia with RTX between 2 weeks and 6 months, suggesting the more potent agent has fewer non-specific toxic side effects. The specificity of RTX is further supported by preservation of motor and mechanical sensitivity, as demonstrated in this study (Fig. 5) and previously [9]. This is not a feature of studies performed with higher doses of RTX which reported deficits in mechanical endpoints [24].

Based on our study with intradermal application of RTX, we selected a dose range for RTX from 25 – 250 ng [8] and evaluated these doses for analgesia and potential side effects. We found that for perineural application of RTX there was a steep dose response relationship occurring between 62.5 and 125 ng, with doses ≥ 125 ng able to significantly block the hyperalgesic response to inflammation (Fig. 3). We used the 250 ng dose of RTX throughout the remainder of the study to reduce variability in responses at the lower doses. In the absence of concurrent inflammation, we observed that animals were able to respond normally to noxious thermal stimuli (Fig. 2). We also noted that there were no obvious effects on the edema produced following carrageenan inflammation when pretreated with RTX, as compared to vehicle. While the neurogenic component of inflammation was eliminated, there are other signs of inflammation such as edema that suggest other inflammatory pathways are not suppressed by RTX. While this animal model suggests that it may not show efficacy in reducing mechanical allodynia, this should not exclude the potential therapeutic action in human pathological pain states, since some of these pain states can be maintained by peripheral inputs. Others have shown that higher doses of RTX given in a variety of routes are effective for reducing nociception [7,24]. Kissin *et al *described a more broad-spectrum effect of RTX administered percutaneously to the sciatic and saphenous nerves and showed decreased sensitivity to normal and inflammatory mechanical and heat hyperalgesia [24]. However at the doses used in that study (1,500 ng), systemic or non-specific effects of RTX cannot be ruled out and may confound the utility for translation into clinical pain control. Recently this group demonstrated reduction of incisional post-operative pain with perineural treatment of the sciatic and saphenous nerve using a lower dose of RTX (450 ng)[25], supporting our contention that smaller amounts of RTX are sufficient for pain control.

Thermal sensing channels, in particular TRPV1, provide targets for discovering new pain therapeutics [34,35]. The use of TRPV1 agonists such as RTX represents an exciting approach for management of pain clinically especially using site-directed application methods [8,9]. In the current study, we found that an anatomically directed perineural application of RTX, blocked inflammatory hyperalgesia, while sparing normal somatosensory input, including thermal and mechanical modalities. As such, localized application of RTX has the potential for a range of uses in pain management, from acute post-operative care to treatment of regional pain disorders.

## Competing interests

The author(s) declare that they have no competing interests.
